# Conceptualising Surgical Innovation: An Eliminativist Proposal

**DOI:** 10.1007/s10728-019-00380-y

**Published:** 2019-07-20

**Authors:** Giles Birchley, Jonathan Ives, Richard Huxtable, Jane Blazeby

**Affiliations:** 1 Centre for Ethics in Medicine, University of Bristol, Canynge Hall, Bristol, UK; 2 Centre for Surgical Research, University of Bristol, Canynge Hall, Bristol, UK

**Keywords:** Ethics, Surgical innovation, Conceptualisation, Research, Governance, IDEAL framework

## Abstract

Improving surgical interventions is key to improving outcomes. Ensuring the safe and transparent translation of such improvements is essential. Evaluation and governance initiatives, including the IDEAL framework and the Macquarie Surgical Innovation Identification Tool have begun to address this. Yet without a definition of innovation that allows non-surgeons to identify when it is occurring, these initiatives are of limited value. A definition seems elusive, so we undertook a conceptual study of surgical innovation. This indicated common conceptual areas in discussions of (surgical) innovation, that we categorised alliteratively under the themes of “purpose” (about drivers of innovation), “place” (about contexts of innovation), “process” (about differentiating innovation), “product” (about tangible and intangible results of innovation) and “person” (about personal factors and viewpoint). These conceptual areas are used in varying—sometimes contradictory—ways in different discussions. Highlighting these conceptual areas of surgical innovation may be useful in clarifying what should be reported in registries of innovation. However our wider conclusion was that the term “innovation” carries too much conceptual baggage to inform normative inquiry about surgical practice. Instead, we propose elimination of the term “innovation” from serious discourse aimed at evaluation and regulation of surgery. In our view researchers, philosophers and policy-makers should consider what it is about surgical activity that needs attention and develop robust definitions to identify these areas: for our own focus on transparency and safety, this means finding criteria that can objectively identify certain risk profiles during the development of surgery.

## Introduction

Innovation in surgery is not always a good thing. Surgical innovation (SI) can produce harms as well as benefits. There is, therefore, a moral imperative to ensure that SI brings optimal benefit with minimal harm. There is widespread agreement that mechanisms providing greater oversight of SI should be developed [36]. This implies that innovation requires appropriate *evaluation* and that evaluation requires appropriate *governance*. Evaluation of innovation needs to be properly designed, conducted and reported to produce reliable high-quality evidence. “Governance” can take various forms, including legal regulation or monitoring arrangements. These aim at ensuring that innovation appropriately balances benefits and harms, to which patient information and consent for receiving an innovative (rather than a tried and tested) treatment can be calibrated. Although they are separate, evaluation and governance have been linked through guidance, notably the IDEAL framework, which sets down ways of evaluating innovation and specifies the types of studies required at different points in the innovation process for evaluating the innovation in question [26]. Yet, evaluation and governance arrangements, whatever form(s) these might take, first require us to grasp what counts as “surgical innovation”. Existing definitions of SI are often ad hoc, vary widely, rarely specify how their elements might be operationalised, and fail to make explicit their underlying assumptions. In this article, we survey and problematise existing notions of SI, ultimately concluding that it is advisable to do away with the term in this context, replacing it with language that better specifies our aims.

Divergent accounts of what SI *is* (Table 1) invite rigorous conceptualisation of SI.^1^ Importantly, we distinguish conceptualisation from definition. Briefly, a definition depends on the normative presuppositions of the defining party and the purposes for which they aim to use the definition, whereas a conceptualisation attempts to map out underpinnings that (largely) transcend specific aims or agendas. Concepts may thus encompass numerous possible definitions, and conceptualisation may produce greater insights into the possibilities and commitments that use of that concept entails than would a simple definition.

The research documented here takes place within a larger project that seeks to improve the safe translation of SI to everyday practice.^2^ The project is undertaking a number of discrete studies aimed at understanding and developing evaluation and governance of SI. To aid these studies we sought to develop a working definition of SI. We reasoned that a conceptualisation would clarify the conceptual areas that a definition might need to consider according its specific aim, and thus could be valuable for others with different aims. We therefore undertook a Critical Interpretive Synthesis [18] to see what this revealed about the concept.^3^ Our synthesis of scholarly discussions of innovation revealed five ‘conceptual areas’ of SI. We term these “purpose”, “place”, “process”, “product” and “person”. In line with our initial aim, our research presents these conceptual areas here as a conceptual toolkit, giving examples of the ways in which they might be used (by ourselves and others) to define SI (in accordance with particular aims). However, we believe our main finding, and that which our analysis shows most clearly, is that the concept of innovation is cluttered with elements that may be unhelpful and obfuscating when attempting to define innovation for governance purposes. We therefore suggest that an eliminativist approach—where the term SI is avoided in the evaluation and governance of surgery in favour of more precise and definite terms—be taken in the bioethical-surgical discourse.

**Table 1:** Examples of current definitions of surgical innovation

Definition	Source
“A novel procedure, a significant modification of a standard technique, a new application of or new indication for an established technique, or an alternative combination of an established technique with another therapeutic modality that was developed and tested for the first time”	[64]:793
“A new or modified surgical procedure that differs from currently accepted local practice, the outcomes of which have not been described, and which may entail risk to the patient”	[15]
“Departures from standard surgical practices that are both nonvalidated and major”	[52]: 607
Anything that sits in the transition zone between practice variations and experimental research	[73]
“A dynamic and continuous process involving the introduction of a new technology or technique that initiates a change in clinical practice”	[29]:205
“A procedure that includes at least one of the following: (i) a different risk profile from standard practice, (ii) the need for new training, (iii) the use of a different anatomical approach, (iv) the potential for increased cost and (v) outcomes that have not yet been described”	[39]:89

## Background

SI has the potential both to benefit and to harm health and wellbeing. Historically, innovations like anti-sepsis and anaesthesia have transformed surgery from an intervention that killed more often than cured, to a speciality that has dramatically improved health [22]. Innovations within heart surgery expanded the borders of survivable disease [12]. Others, like endoscopy, have increased wellbeing and lowered the risks and costs of surgery by removing the need for large incisions, which improves cosmesis and reduces recovery times [37]. Contemporary innovations in technique, like natural orifice surgery, may build on these developments [54], while devices used for robotic surgery potentially raise the aptitude of surgeons undertaking minimal access techniques to elite levels [14].

Despite such positive developments, however, many innovations fail to deliver on their apparent promise. Chymopapain chemonucleolysis [79], jejuno-ileal bypass [54], and power morcellation of uterine fibroids [21] are among many widely adopted SIs abandoned because of their deleterious effects. Contemporary innovations, such as vaginal mesh in gynaecology, may yet join this list [27].

Failed innovations can damage the lives of patients, their loved-ones, and the reputation of surgery and surgeons. We suggest improving the safe translation of innovations is an ethical and a pragmatic imperative. One way to do this is to improve standards of reporting in surgery by identifying innovation when it occurs. The IDEAL Collaboration [31] has taken steps toward this goal. Based on a translation of the preeminent theory of diffusion of technological innovation [68] to surgery [79], the IDEAL framework divides the development and diffusion of SI into five stages. At each stage the innovation affects more patients, triggering a regulatory model and outcome measure (Table 2).

**Table 2:** The IDEAL framework. Adapted from Lee [39]

IDEAL stage	Stage 1 (idea)	Stage 2a (development)	Stage 2b (exploration)	Stage 3 (assessment)	Stage 4 (long term study)
Number of surgeons	Very few	Few	Many	Many	All eligible
Number of patients	Single to few	10s	100s	100s +	100s +
Ethical oversight	Informed consent only	Register protocols, local ethical approvals	Standard research ethics approvals	Standard research ethics approvals	Informed consent only
Outcome measurement	Case reports	Prospective development studies	Feasibility randomised control trial	Randomised control trial or alternative designs	Registry, audit

The IDEAL framework provides a template for regulating the development of innovations. Improvements to IDEAL have been proposed [9], including those relating to its definition of innovation. IDEAL defines innovation as “a new or modified surgical procedure that differs from currently accepted local practice, the outcomes of which have not been described and which may entail risk to the patient” [4]. Critics claim this definition is overly broad [40], fails to distinguish between variation and research, and lacks practical utility [70]. Since correctly identifying when innovation is occurring is central to effective oversight, this is potentially a serious limitation [33].

The Macquarie SI Identification Tool (MSIIT) addresses this limitation by operationalising a definition of innovation based on ‘newness’ [30]. MSIIT encourages surgeons to subjectively judge if they are innovating by considering whether the procedure/device is new, where ‘newness’ is defined by reference to the procedure/device’s difference from standard practice.^4^ The tool identifies relevant categories in which newness may occur. These are in relation to a device, tool or technique, and in each of these categories, may relate to use in patient groups, for indications, and in anatomical locations not previously associated with that tool, device or technique. The tool has a supplementary checklist including questions about prior evidence, publishability and preparation, all of which are also intended to help surgeons identify newness in their own practice.

That MSIIT is based on valuable conceptual research that sets it apart from previous ad hoc definitions. Nevertheless, the tool itself is open to four criticisms. First, using ‘new’ to define innovation appears to swap one indefinite term for a related indefinite term, since ‘Innovation’ is etymologically closely related to ‘new’.^5^ Although this takes place at a high level and further specification follows, it is not clear what is gained by using the term ‘new’, rather than ‘innovation’, other than a change of terminology that seems vulnerable to some version of the Open Question Argument (i.e. it would still seem meaningful to ask ‘yes it’s new, but is it innovation?’). Second, “no technology or its application is entirely new, as no inventor works within a vacuum” [67]. Innovation may involve only small, incremental differences to normal practice [4, 59]. Even radically innovative devices and procedures are likely to have established components. What constitutes “newness” is subjective and susceptible to manipulation.^6^ While MSIIT attempts to limit this malleability by focusing on specific categories of newness, within these categories newness remains subjective, limiting the value of MSIIT to *governance*. While MSIIT seeks to clarify newness with examples, this obscures the question of whether newness is an effective criteria for line drawing. We suggest it is easy to circumvent. Third, subjective newness is intentionally broad, so the tool measures many false positives and relies on the surgeon to identify which cases are mistaken. This passes the burden of identifying innovation to the surgeon (by asking them instead to identify what is new), thus undermining MSIIT’s aim of *aiding* practitioners. Finally, and importantly, it is questionable whether surgeons are the most appropriate people to judge innovation in their own practice. Research identifies surgeons as subject to various conflicts of interest [34, 44, 69]. Moreover, there appears to be little consensus about what constitutes innovation among surgeons [66, 70]. One strategy being pursued by the Macquarie team [71] to mitigate this final criticism may be to ask theatre teams, rather than individual surgeons, to use MSIIT. Yet there remains a serious risk that team dissent will be squashed by workplace hierarchies that give a surgeon the final word.

Helping surgeons to identify innovation in their own practice appears laudable, as this could encourage the safe translation of innovation to practice by encouraging effective evaluation studies. However, MSIIT is an incomplete response as, practically, we still require definitions—for effective governance and beyond^7^*—*to objectively identify innovation. For this reason, although indebted to the conceptual work that underlay MSIIT, we wished to step back from the MSIIT definition. We intended a conceptualisation of SI that transcended specific aims and agendas, to uncover the possibilities and commitments involved in formulating a variety of definitions to meet a variety of aims. Our approach to conceptualisation is discussed now.

## Methods

While sometimes considered the foundation of philosophy, conceptualisation is a contested field [41]. Classical approaches to conceptualisation suggest that concepts comprise separately necessary and jointly sufficient features [32]. Yet, classical approaches to conceptual analysis have produced few (if any) satisfactorily defined concepts [80]. Experimental psychology indicates that the way humans approach concepts is not explicable using classical approaches. Instead, numerous studies indicate that some examples within a conceptual category are considered more typical of that category than others. For example, ‘sparrows’ are more readily categorised as ‘birds’ than ‘penguins’ [19]. Many agree [41, 43] that concepts are not definitions.^8^

### Approach to Conceptualisation

While concepts are not definitions, conceptualisation can inform the development of definitions. Current paradigms, derived from psychology, suggest that concepts comprise hazy and overlapping features, none of which inevitably embody universally necessary and sufficient features.^9^ No candidate theory of concepts seems completely adequate,^10^ prompting radical reassessments of conceptualisation that include proposals to abandon concepts or consider concepts in pluralistic, rather than monistic, terms [41, 63]. In our view, the hazy, overlapping features that make up a concept mean that a concept can underwrite numerous, sometimes conflicting, definitions. Conceptualisation can identify features of a concept, but further (including normative) work is then needed to determine which of those features can be brought together to form a definition for a specific purpose. Different needs, goals and normative presuppositions will result in different definitions, which bring together different features of a concept.^11^ We hypothesised, therefore, that it should be possible to conceptualise SI as features of SI *qua* SI, and draw on these features when developing future definitions for specific purposes. Our idea was that such a conceptualisation should contribute some underpinning structure to the different definitions of different policy-makers, researchers and practitioners. By undertaking a study of the ways SI, and innovation more broadly, is discussed by commentators in the literature, we intended to provide such a conceptualisation.

Our investigation began by synthesising the ways innovation is discussed in surgery and a range of other disciplines. A conceptualisation so derived elucidates conceptual areas of SI, but we reiterate that this was not to furnish a definition by providing the necessary and sufficient features of SI. Instead, our intention was to detect an undergirding conceptual structure.

### Synthetic Method

Primary sources in the study of innovation are found in agricultural sociology and economics. Application of the concept to surgery brings in literature from not only surgery, but also bioethics, health policy and medical history in which SI features. As such, our method needed to sample literature from numerous fields of inquiry. We adopted Dixon-Woods et al.’s [18] Critical Interpretive Synthesis approach, which takes a non-linear, iterative method of reviewing and analysing literature (Table 3).

**Table 3:** Critical interpretive synthesis. Adapted from Dixon-Woods et al. [18]

Although non-linear and iterative, critical interpretive synthesis could be understood to follow these steps	
Step	Sub-step
1. Using a broad, provisional research question, undertake a literature search	
2. Gather identified papers into a sampling frame	
3. Iteratively select sources from the sample frame, review and extract data	(a) Sources are purposively selected from the sample frame using principles of theoretical sampling (e.g. contribution to research goal); fatally flawed studies are discarded
	(b) Extract data using the thematic method to develop codes for key arguments and phrases and theories; cluster codes into themes
	(c) Continue theoretical sampling, data extraction and theme development until theoretical saturation
4. Synthesize arguments from the themes, integrating these with existing evidence	

**Fig. 1:**
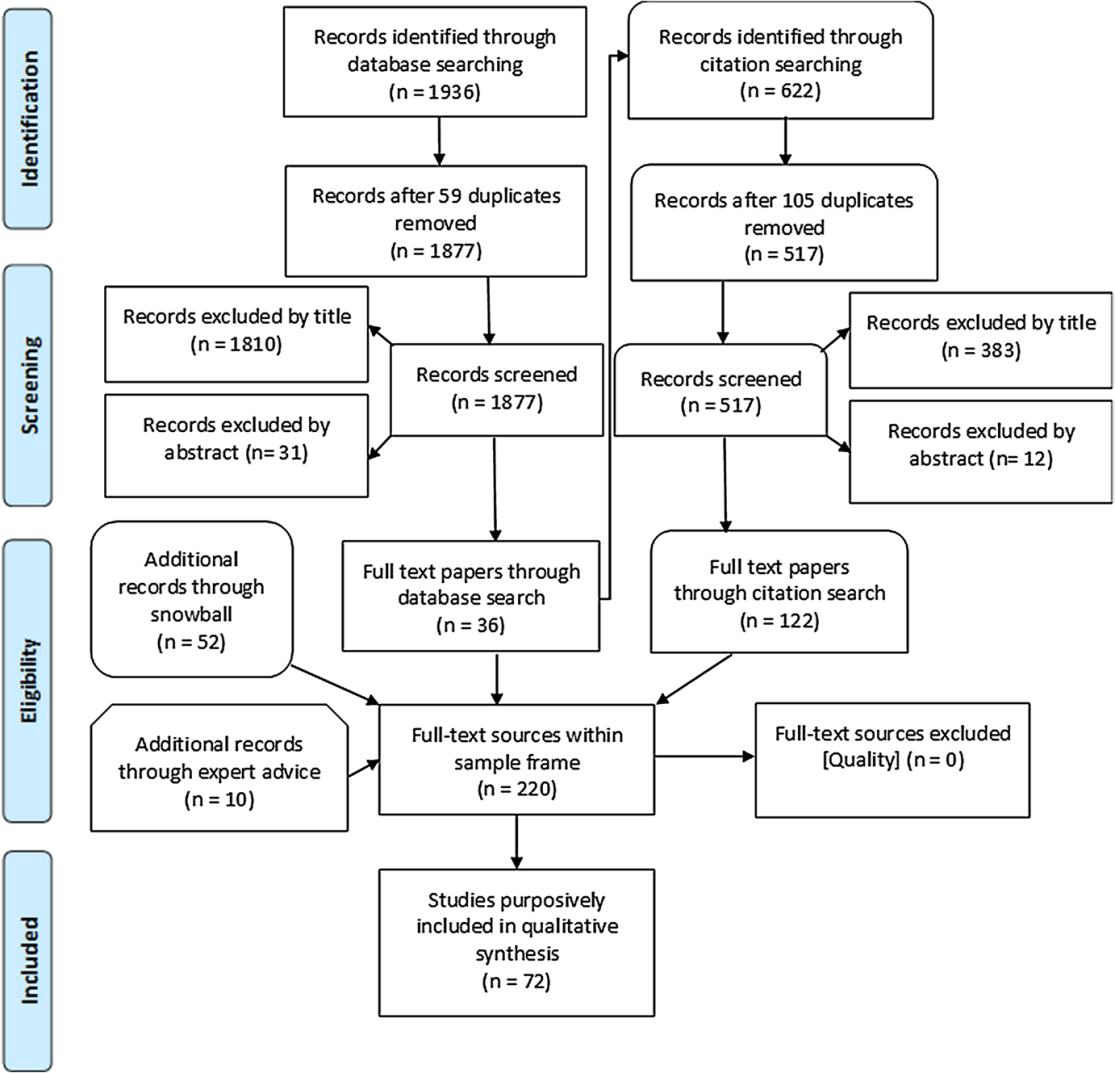
Literature search flowchart

Using keyword searches derived from the initial research question “How is innovation and its phases (e.g. ‘evolution’, ‘stabilisation’, ‘adoption’, ‘abandonment/rejection’) conceptualised and articulated, as they pertain to surgical innovation/innovation in invasive interventions?” we derived a sample frame of 220 sources. Our initial searches excluded clinical research articles reporting surgical outcomes, reasoning these would not allude to the concept of innovation in a form amenable to analysis. Excepting this criteria, any sources that offered reasoned discussion of what innovation is were included (see Fig. 1).^12^

Analysis followed the principles of theoretical sampling. A total of 72 sources^13^ relevant to the developing line of enquiry were purposively selected from the sample frame and coded using the thematic method [11]. Purposive selection treated individual sources as research informants. Individual sources were selected because their title and abstract appeared to inform the emerging questions as they were perceived by the authors at the time: thus we were guided at different times to, for example, bioethical accounts, practice descriptions, innovation theory and historical studies. The sample discussed, and sometimes defined, innovation, but for the most part made no specific claim about the content of the concept of SI. Indeed, even where sources did make such claims we did not take such assertions merely at face value. Instead we took a critical approach and coded all parts of the discussion that pertained to the nature of innovation to reveal the structure of the underlying concept without imposing any further normative screen on the results. We reasoned this was a legitimate interpretation of the Critical Interpretive Synthesis approach, which we had adopted precisely because of the indistinct nature of SI and the numerous competing definitions and claims that surround it. Throughout the analysis, all authors discussed the developing codes, areas needing additional investigation, and outcomes of analysis.

### Analysis: Developing the Concept

Our study suggests 5 conceptual areas pertaining to SI (Table 4). For clarity, it is worth reiterating here that these conceptual areas do not themselves constitute a definition of any sort. Instead they are facets of a rich—and, we will argue, not entirely coherent—concept of SI. Detailed analysis of these conceptual areas is given below.

**Table 4:** Themes and descriptions

Theme	Conceptual area	Description
Purpose	Drivers of innovation	Where does innovation come from, to what should innovation respond?
Place	Context of innovation	What is the relationship between innovation and research, early adoption and routine variation and/or it’s geographical place?
Process	Differentiating innovation	How does the innovation depart from standard approaches or outcomes?
Product	Consequences of innovation	What are the (in)tangible results of the innovation?
Person	Identity of innovator	Who is the innovator, what are their character traits and intentions?

#### 1. “Purpose”: Drivers of Innovation

Studies explored the drivers of SI, suggesting that SI may be undertaken due to patient, surgeon or industry needs or desires. This category also raises the question of whether demand is created by innovations or vice versa.

*Responding to Patient Needs or Wants* The interests of patients, surgeons and industry may to some extent overlap, making clear distinctions about what drives innovation is problematic. Nevertheless, the literature identifies a number of different drivers. Some suggests that patient wants and needs are drivers of SI [7]. These motivations encompass usually bifurcated positions in economic analyses of innovation. Some economists see demand for innovation as being created by the innovator, in what is termed “technology push” [28]. Others see innovation as a response to external or public demand [58]. Likewise, wants and needs can arise from the patient or be identified by the surgeon (who we consider in due course). In relation to patients, wants and needs are (sometimes) distinct: a want will exclusively arise from the patient, whereas a need may be identified by the patient or a third party. Barkun et al. consider patient need to often lie at the root of SI [4], a motivation expressed by surgeons themselves [15]. Meyerson argues that patients who demand it have a right to innovative surgery in terminal illness if there is some evidence of effectiveness [53], which suggests innovation satisfies wants. Wants and needs need not be distinct, although they are often ranked, with needs putatively taking priority over wants in emergency treatments [35].^14^ Outside the bounds of emergency experimental treatment, wants may take precedence. Wants are often identified in discussions of medical innovation. Public demand, a corollary of want, is identified as a driver for medical innovation in some analyses [1, 6, 51]. McKinlay argues that public demand, stimulated by media reports, places pressure on funders to ensure that innovations are widely available [49]. On this reading, public demand plays a role in the diffusion of medical innovation, but the innovation originates prior to this demand.

*Surgeon and Industry Demand* In surgery, demands may come from patients, industry, or arise from surgeons. Surgeons may want to perform a procedure more efficiently or quickly in order to meet their own or institutional need, or they may want to build their career as an ‘innovator’—and this may or may not coincide with responding to patients’ needs. The introduction of innovative devices appears largely driven by industry, based on a desire for market share, and can be imitative of existing devices [34, 59]. Such analysis suggest that it could be the wants of industry or surgeons that are served by these technologies, with public need a peripheral (or even absent) focus. It is possible therefore that neither the wants nor the needs of the public are directly important to SI, but that SI plays an economic or career-enhancing function. Some suggest there is something morally problematic about this orientation [66],^15^ which may provide a reason to define innovation in a particular way for the purpose of governance.

In summary, the role of wants and needs of various parties suggests that “Purpose” is a conceptual area of SI. Definitions of SI may thus take a position on how—if at all—the drivers of an activity feeds into whether it is treated as innovation or not, and why this is the case.

#### 2. “Place”: Context of Innovation

The concept of SI also pertains to *where*—contextually and geographically—innovation is held to take place. Debates about context largely turn on the relationship between innovation and research, with a subsidiary debate about whether premeditated variations constitute innovation or research.

*Geographical Place* Discussion of geographical place is limited to whether diffusion of a SI to a new a location should [4] or should not [30] play a part in identifying SI. Studies demonstrate isolated instances of adoption may occur when a procedure is well-established within surgery at large [20].^16^ A learning curve is associated with the journey from novice to expert in a particular procedure [24]. The learning curve results in relatively poor performance when an innovation is first adopted [12]. A new geographic location that entails changes of personnel therefore impacts on patient safety, which may be an important focus for oversight when defining SI. If safety is less important, it is conceptually simpler to discount new locations from a definition of innovation. Tying innovation to first time use of all or part of a procedure captures each first time use of established procedures, identifying all early and late adopters as innovators in a way that could problematically impact on governance by inundating governance structures with low-risk cases and burdening practice with disproportionate levels of scrutiny.

*Different to Research?* As a contextual feature, whether innovation is or is not distinct to research is extremely important. If it is indistinct, then the label of innovation is a mere flag of convenience to avoid scrutiny. If it is not, then it represents a lacuna that is insufficiently considered by current governance structures. The literature is divided on whether innovation is equivalent to, or distinct from, research. While acknowledging that research may follow innovation, many distinguish research activity from SI [2, 3, 16, 21]. Others consider SI to be research [40, 52, 65].^17^ Some accept the ambiguity of one or both terms without drawing any firm conclusions [10, 44].

If the status of innovation *vis* research is ambiguous, whether an activity is labelled by a surgeon as innovation or research is merely a choice of terminology and may be driven by a desire to avoid the burdens accompanying research [40] or access benefits accompanying ‘innovation’.^18^ These burdens differ between jurisdictions: for example, U.S. insurers will not pay for ‘experimental’ therapies [44],^19^ while the UK government encourages ‘innovation’ [17].^20^ Some argue that existing definitions of research (which emphasise generalisable theories and principles, scholarship and hypothesis testing) ill-describe innovation [40, 45]. Indeed, rather than hypothesis testing, some associate innovation with hypothesis generation [50]. Extending the idea that an innovation is performed specifically to benefit a single patient, some argue that innovation does not produce generalisable knowledge [35, 73]. However, as knowledge gained from each patient encounter is likely to be used in others, this also appears a shaky distinction. Other popular definitional criteria for research are also used to distinguish innovation [13]. Spontaneity allegedly distinguishes innovation from research, which is presumed to be premeditated [67, 73]. Some argue that surgical practice frequently necessitates ‘routine variation’, where spontaneous changes in practice take place in response to anomalies in patient anatomy [8]. These authors, like the surgeons in a survey by Reitsma and Moreno [66], suggest that routine variation is a feature of normal surgical practice, not research. Attempting a paradigm shift, Lotz argues that this (and other) intrinsic features of surgery make *all* surgery research [40].

The relationship of SI to research, routine variation and standard practice remains contested. If safety issues inform whether something is defined as innovative, then that definition may need to attend to many other aspects, such as the learning curve, and thus geographical place. Definitions may also consider ‘place’ in terms of context and, depending on the aim of the definition, either choose a definition that disentangles innovation from research or a definition that makes innovation part of a research process. Any of these options might be justified but will imply different kinds of governance.

#### 3. “Process”: Differentiating Innovation

The concept of SI includes consideration of *how* innovation is achieved, which may be characterised as a process, a discrete event, or combinations thereof. SI may, therefore, involve a one-off event or a series of events (and revisions), with the latter resembling a process. However it is achieved, if the process/event of SI is to be distinguished, it must have features, like impact or newness, that mark it as different from the process/event of normal surgical practice.

*Nature: Process or Distinct Event?* Perhaps due to their differing aims, the MSIIT and IDEAL implicitly take different approaches to the development of SI. MSIIT, in searching for criteria that can prospectively distinguish routine variations and introduction of new procedures to new geographical locations from SI [30], presuppose a distinctive timepoint where innovation occurs. IDEAL, by adapting an existing model of technological innovation to SI, focuses on an ongoing process of SI that includes development and diffusion of the innovation into the mainstream. That this dichotomy is most credibly due to the differing aims of MSIIT and IDEAL is borne-out by comparing other areas where it occurs. While not always considering SI (rather than medical innovation more broadly), the distinctive aims of legal treatments of innovation mean they tend to focus on distinct time-points of innovation [13, 38], while attention to the learning curve and the spread of innovation in the surgical literature (in general) means that innovation tends therein be considered a process [24]. While unintentional, studies indicate epistemic costs to both approaches. A model of innovation based on process implies “more order and coherence exist than is actually the case” [49]. Similarly, conceiving innovation as an event fails to account for the relationship of imitation, reinvention and iterative change to innovation. These relationships seem important: Schumpeter’s economic theory asserts that successful innovations result in imitation [28]. Rogers’ sociologically based theory of innovation views reinvention as part of the dynamics of adopting technological innovations [68]. Descriptions of SI arising from the IDEAL framework, including within (rare) descriptions of developing a SI [75], suggest iterative changes play an important role in the process of innovation [4]. Iterative change is also germane to the notion of ‘enabling technologies’*—*existing innovations whose dissemination allows further innovations to arise [67]. Iterative change suggests that the line between producing and adopting a SI may be fuzzy, and underlines the point that the way we choose to define innovation will involve both gains and losses to clarity [49]. Moreover, how iteration of innovation is conceived may affect the level of governance that the innovation receives. For example, Schwartz proposes that a single instance of innovation should not require oversight, but repeated instances should [73].

*Features: Departing from Existing Standards* The literature generally argues that SI must differ from what occurs in surgery on an everyday basis. The 1978 Belmont report on research ethics suggests that medical innovation occurs “when a clinician departs in a significant way from standard or accepted practice” [57]. This type of formulation is commonly repeated.^21^

One frequent way this difference is characterised is as ‘newness’, or similes such as ‘novel’ or ‘first’ [21, 30, 48, 73]. As noted above, classifying newness is subjective. All new surgical procedures will be composed of basic techniques (e.g. dissection) which are not themselves new. Moreover, depending on whether iteration is included in a characterisation of SI, new techniques will (perhaps always) build on previous ones, creating an area of discretion between modification and ‘absolute’ newness. Additional terms may address this imprecision. For example ‘new combinations’, was proposed by the economist Joseph Schumpter as a loose, catch-all definition of innovation [72], and this approach has been utilised in some definitions of SI [21, 30, 44, 64]. Other discussions use terms of magnitude such as ‘major’ or ‘significant’ [56, 64] to avoid too “broad and blunt” a definition of innovation [40] Hutchison et al. caution that such modifiers are ambiguous and should only be used with clear guidance about their intended interpretation [30]. Thus we can see a tension between inclusive and exclusive modifiers: every extra modifier threatens ambiguity, yet each absent modifier increases the chances that a definition will be overly broad and thus swamped with positives.

Within this conceptual area, thought must be given to whether a definition aims at breadth or precision, and to which compromises to accuracy are acceptable. Defining SI as a process may exclude details to increase intelligibility and simplicity, while a definition that favours a distinct event must be clear about how iteration is explained and/or excluded.

#### 4. “Product”: Consequences of Innovation

The concept of SI includes any products of SI. Narrowly construed this product may simply be the surgical procedure or instrument itself. However, commonly the products of an SI include (some or all of) the consequences of the innovation. Consequences might be tangible, like patents, or intangible, like risk. To some degree a discussion of consequences may overlap a discussion of the drivers of innovation (our theme of “Purpose”) as particular consequences may be assumed in embarking on innovation. Nevertheless, we feel that consequences are more coherently considered as a theme in their own right,^22^ and the overlap here reinforces the aptness of Wittgenstein’s analogy between a concept and a rope comprising multiple overlapping threads.

*Tangible Products* Tangible products, such as patent or the prospect of publication have been associated with SI [8, 29], including by surgeons themselves [70]. Since patent/publication will tend to exclude failed innovations, focus on these products supports the view that SIs are, by definition, successful [7]. Moreover, at least in the case of patent, this employs a somewhat circular reasoning that a legal framework for patenting can be a shortcut to defining innovation, which we feel reads too much certitude into the patenting process. A second class of tangible products are socio-economic impacts. In economics, Schumpeter defined innovations as necessarily disruptive of the current economic equilibrium [72], Socio-economic impact of SI is not uniform, and some authors distinguish between the most impactful (disruptive) and least impactful (incremental) innovations [21, 30, 67]. Arguably, identifying disruptive innovations is important to definitions aimed at historical, economic or social evaluation of SI.

*Uncertainty* Intangible products such as risk are commonly associated with SI [8, 40, 73].^23^ Although Rogers et al. argue that innovations may sometimes reduce risks [70], new occurrences that diverge from the norm introduce uncertainty, suggesting that risk*—*of some magnitude*—*may be a persistent feature of SI. Discussions often view risks as proxies of harm, however the flip-side of this is to argue that benefit is the defining product of SI. This benefit may be characterised abstractly or indirectly*—*such as in terms of producing knowledge [40, 56].^24^ Alternatively, benefit may be characterised in specific and direct terms, for instance benefiting a particular patient who undergoes the SI [70]. While benefit may suggest more confidence in the product of the SI (and thus less unpredictability) than risk, uncertainty remains. When and where benefits or risks transpire relates to the account adopted about the process of SI. If SI is a distinct event, the benefit or harm might also be a distinct event, while, on a process account, several intended benefits or harms may occur at distinct phases in the career of the same SI. Importantly, not all ‘innovative’ products may produce any risks or benefits. Some commentators are sceptical of the presumption that medical innovation does or will produce benefits on every occasion [49], and benefits are not included in wider definitions of technological innovation [68]. Similarly, innovation need not introduce extra risk. Standard business models of surgical instrument manufacturers involve the production of so-called ‘me-too’ products [59].^25^ Arguably, these minor modifications may give a (patentable) impression of difference without changing the risk profile. Nevertheless, even minor differences will introduce new uncertainties (for example, just changing the colour of an instrument might cause it to be misrecognised). The conviction that the products of SI are unpredictable unifies these perspectives.

While consequences are by their nature unpredictable, some observe that risks and benefits of SI cannot be known prospectively; [30] indeed, decades may elapse before such information is completely clear [12]. Others suggest that risks of SI may nevertheless be reasonably estimated, for example by considering similar, established procedures [54]. Indeed, some argue that, rather than actual outcomes, differences in *expected* outcomes are markers of innovation [73]. A definition of SI intended to be prospectively applied might therefore exclude consequences from a definition of innovation, as (at least partially) does MSIIT, or find an acceptable way of prospectively estimating risks and benefits. A concept of SI includes any tangible/intangible products that arise from SI, including socio-economic impact, benefits and risks. Definitions employing such terms require appropriate methods for assessing these impacts to be identified. Clearly tangible products are easier to measure in this respect. How definitions approach “Product” will be affected by their intended use: for example, whether they identify SI prospectively or retrospectively, intend to enhance patient safety, underwrite patentability or recognise socio-economic events. Because of the prominence of unpredictability in this conceptual area, the risk profile will arguably feature heavily in definitions aimed at enhancing patient safety, but may be less important for other purposes such as identifying historical innovations.

#### 5. “Person”: Identity of Innovator

The identity of the innovator in SI is conceptually significant given the prevailing model of innovation (on which IDEAL is based), and the frequency with which surgery and surgeons are identified as innovative. Further, the conceptual area of ‘person’ includes questions of intention central to whether definitions identify SI as subjectively or objectively determined.

*Personal Characteristics* Rogers’ influential sociological theory of the spread of technological innovation explicitly categorises innovators and adopters according to personal characteristics. These include those related to socioeconomic status and “personality variables” including empathy, rationality, and intelligence that Rogers collectively terms ‘innovativeness’ [68]. Rogers’ theorises that the most innovative members of any population (“innovators”) comprise an elite 2.5% of a target population [68]. Rogers’ laudatory approach to innovator character is similar to Schumpeter’s “heroic” risk-taking entrepreneurs in economics [28]. Emphasis on positive innovator character has been questioned in agricultural sociology [77], however the surgical literature frequently asserts the special personality of innovators and/or surgery as an intrinsically innovative discipline. Some repeat Rogers’ suggestions about personal characteristics in the context of SI [14]. Others identify surgeons’ personality traits [47] including affinity to new technology [67], compassion [64], conscientiousness [52] and dedication to science [69]. Surgical culture claims a unique affinity between surgeons and innovation [44]. SI also has a putative relationship to expertise. Since SIs are claimed to be difficult procedures [48], or at least requiring expert knowledge, innovators are often identified as expert surgeons [30, 73].^26^ Nevertheless, innovation is not always synonymous with expertise. Cardiac catherization was innovated in humans by a novice surgical trainee [78].^27^ Historically, Ben-David argues expert scientific cultures have been antagonistic to innovation [5], and some commentators identify the conservative nature of surgical practice as a block on SI [67].

*Intention* The intention of the surgeon is also, often, noted in discussions of SI, underlining the connection of innovation to debates about what constitutes research, where intention is also considered important [13]. Some allege that the intention to benefit a current patient and to benefit future patients respectively distinguish innovation and research [52, 73]. A darker view of intention is taken by Reitsma and Moreno [65] who imply that many surgeons willfully avoid outside scrutiny. Others argue that surgeons are subject to unconscious biases hampering their ability to scrutinize their own intentions [69]. Difficulties objectively identifying intention affect the status of SI as something that can be identified subjectively or by any neutral observer. Much of the current use of SI suggests it is subjectively identified, and MSIIT tends toward this, albeit open to an intersubjective, team-based, approach [30].^28^ Subjective determination of innovation raises the potential for independent origination of similar innovations. Because this may result in two surgeons unnecessarily performing the same risky SI, taking innovation to be subjectively determined requires strong safeguards to prevent unnecessary (and hazardous) replication. Such a conceptualisation of innovation thus appears as a strong motivator to establish registries of innovation—a widespread aspiration in the literature [40, 62, 73]. Laissez-faire approaches to the regulation of SI predominate in the literature [36] and defining SI subjectively provides a strong impetus to this laissez-faire approach. Ultimately, the specific aims of the defining party will determine how this conceptual area is addressed. Definitions aimed at protecting current surgical practice may define SI subjectively, while those aiming to alter practice may define SI using a more observer-neutral approach.

As with the preceding areas, the approach to this conceptual area will be guided by the aims of a definition. Definitions aimed at sociological studies of surgeons may concentrate on personological descriptions of surgeon innovators, while definitions aimed at safety may seek define innovation objectively and independently of the person.

## Discussion

The analysis we have presented from our Critical Interpretive Synthesis has two distinct uses that we will consider in two main sections in this discussion. In the first we offer a conceptual structure for future definitions of SI. The second section discusses our increasing scepticism about the usefulness of SI as a concept, that has led us to question the salience of a definition that claims to identify every instance of SI. Thus, the second section of our discussion presents our analysis as evidence that future studies should take an eliminativist approach to identifying SI. By this we mean that, in inquiries that investigators intuit to be about SI, rather than attempting to define SI, the more important and useful task is to determine what role the term ‘innovation’ would play in one’s inquiry, and then to devise specific definitions that identify the surgical activity pertaining to that area. Our broad motivation for this conclusion is that the term SI appears to carry a great deal of ambiguous conceptual baggage that can only serve to obfuscate and avoid scrutiny of the development of surgical practice.^29^ We acknowledge that this is a radical solution with which not all will agree, but which does, at the least, demand reasoned objection. We thus begin with a conceptual structure—or toolkit—that may be useful in devising future definitions of SI.

### A Conceptual Toolkit

**Fig. 2:**
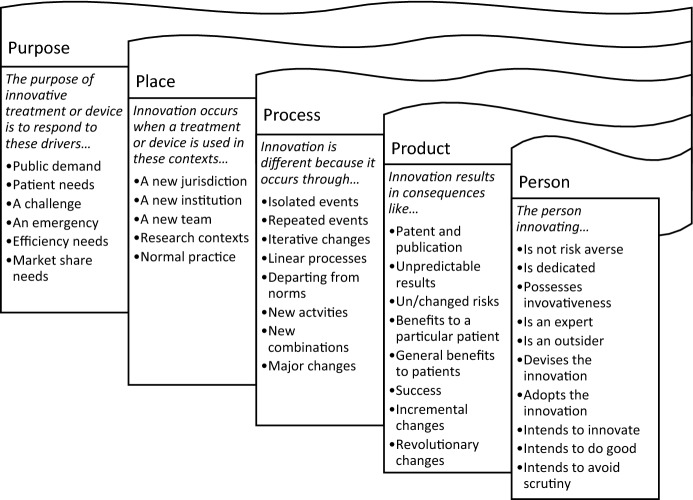
Ways conceptual areas could be resolved

There will never be one single definition of SI, but rather numerous definitions designed for numerous purposes. The five themes of purpose, place, process, product and person relate to conceptual areas that constitute a conceptual toolkit to help ensure a definition is justifiable at a structural level, and carefully considers what it needs from each conceptual area. Each of these conceptual areas require consideration when devising a definition of innovation. A collection of non-exclusive ways that these elements could be resolved into a definition is given below. For clarity we reiterate that these are not intended to be examples of necessary and sufficient features, and we are not proposing a definition. Indeed, some of the elements would be contradictory if taken together. Rather, in Fig. 2, we illustrate a range of possible claims that could be made under each conceptual area, and which might be selected from to build a definition of SI.^30^

Potentially such a toolkit could inform what ought to be reported when information about surgical techniques or equipment is shared. Numerous commentators have proposed (sometimes compulsory) formal registries of SI [34, 55, 59, 61]. Registries are widely supported in the literature [25, 39].^31^ Some registries exist, but they are not always well used [39]. Registering innovation presents a practical problem since innovation is not well defined. Presuming some agreed definition of SI is desirable and possible, the transparency and usefulness of entries to a register may be increased by reporting information related to purpose, place, process, product and person.^32^

### An Eliminativist Approach

As we stated at the outset of this article, our primary purpose is improving the evaluation and regulation of surgical practice. While a conceptual toolkit of the sort presented above was our original intention, we were unprepared for the breadth of potential definitions a conceptualisation would support, and this might impact on our primary purposes. The nature of each conceptual category allows diametrically opposing interpretations, and our analysis shows that at least some of these opposing interpretations are apparent in the literature. Further, many of the conceptual areas would not be needed for our own, regulatory, purposes. This again underscored the scope for dissimilar definitions of a putatively similar phenomenon in different disciplines. For instance, the “Purpose” of a SI might be of interest to social scientists, but be of little help in identifying innovation for regulatory purposes. The context variability was particularly true of the fifth conceptual area, “Person”. The frequency with which the various claims of the affinity of surgeons and surgery to innovation and the regularity of commentary that claimed that intention was a key feature of innovation meant that the person-centred conceptual area appeared important to SI. Nevertheless, a focus on the innovator, and the implications this has for the subjective determination of SI by the surgeon, seemed to work against the (to us compelling) arguments for the need for *external* oversight and regulation of SI to ensure patient safety. It is also notable that not all of these conceptual areas had discriminatory power. Despite them being common themes that run through the discussions of innovation we examined, many had much in common with other types of healthcare interventions. We reasoned that such lack of distinction may be because innovation itself is not distinguishable from much of healthcare. Since so much that was needed to conceptualise SI was either not needed for our purposes, seemed to obfuscate the activity we had intended to identify, or was common to healthcare practice, our conclusion is that an eliminativist approach to SI would be more effective. By effective, we mean that an eliminativist approach would reduce the chances that regulation could be misinterpreted (willfully or otherwise) or misunderstood, thus ensuring a more stringent approach to evaluation and regulation focused where (we argue) it is most needed. This approach stemmed from the view that SI was too hopelessly laden with conceptual baggage to be useful in bioethical discourse. By ‘eliminativist’ we do not mean erasing innovation from language altogether—we agree this would be undesirable and likely impossible given such language is entrenched in surgery. “Innovation” will retain some, primarily rhetorical, uses. But we should call out the essential meaninglessness of the term in serious discourse aimed at evaluation and regulation. In these areas, we should consider what it is about surgical activity that needs attention and develop robust definitions to identify these areas. For example, our own concern is that some surgical activity needs to be safer and more transparent. Given that focus, what seems important to us is both the need for surgical activity to be transparent to third-parties, and (lack of) knowledge about the safety and efficacy of a subset of surgical activities (that may otherwise be labelled as SI). This focus suggested our definition should therefore be objective and should not rely on the probity of individual surgeons to self-report. Rather, it should concentrate on identifying how much is prospectively known about the safety and efficacy of a procedure.^33^ This requires analysis of the extent an intervention presents a substantially new risk profile because of its difference from existing interventions, to what extent a risk profile can be anticipated because components of the intervention are tried and tested, and what new risks arise from any hitherto untried combination of these components. Governance should therefore focus on assessing risk, appropriate methods for studying changes to surgical practice according to its risk profile, and the appropriate reporting of outcomes. We can therefore jettison the language of innovation, which by being all things to all people provides cover for both misunderstanding and wilful avoidance of scrutiny, and instead focus on the need to regulate for the reporting and monitoring of any planned or unplanned changes to invasive surgical procedures that result in an uncertain risk profile. This is no small task itself, but it is made simpler now that we have rejected the conceptual baggage that comes with ‘innovation’ and can focus exclusively on what is important to our aims of improving transparency and safety. Our suspicion is that other studies would benefit from the clarity that taking this eliminative approach can bring.

## Conclusion

SI raises numerous ethical and practical questions that suggest it requires greater oversight. Despite the progress that has been achieved in this field, the absence of robust definitions frustrates this purpose. We observe that the definition formulated by the MSIIT study serves specific purposes that make specific (and questionable) assumptions about the probity of surgeons and the nature of innovation. A prospective definition to be used by surgeons is only one of many types of definition that may be useful to the aim of the safe translation of SI into everyday practice. Our conceptual study synthesises a range of sources, concluding that the concept of SI includes information about these conceptual areas; the drivers and purpose for which innovation is undertaken; the contextual and geographical place where the innovation is undertaken; attempts to differentiate innovation from other processes; its tangible and intangible products and consequences, and; the viewpoint and personal traits of the person who innovates. While potentially elucidating definitional criteria, these are so broad as to allow numerous dissimilar definitions of SI. These may further obfuscate and complicate oversight of SI. We therefore, for the purpose of evaluation and governance of surgery, propose an eliminativist approach. Instead of seeking to formulate a universal definition of SI, this approach focuses on the features of the surgical activity that we consider to be in need of scrutiny and develops robust definitions to identify these. Such an approach is both simple and effective, and ultimately will benefit both patients and surgeons.

## Footnotes

1 As we will discuss shortly, our work builds on significant work has already been performed on this topic by a team from Macquarie University led by Professor Wendy Rogers see: [30].

2 The project is the “Surgical Innovation” strand of the NIHR Bristol Biomedical Research Centre. See: https://www.bristolbrc.nihr.ac.uk/our-research/surgical-innovation/

3 A conceptualisation ideally requires engagement with both practice and theory. To the extent that our study incorporated qualitative explorations of practitioners views, we were attentive to this, but we would be the first to acknowledge the dearth of the literature in this area (for example, we know of only a single survey of patients views). Thus we are currently engaged with further qualitative exploration of both practitioner and patient views and experiences.

4 A similar, approach, based on subjectively defined newness was proposed by SAGES guidance on the subject published the same year. See: [76].

5 ‘Innovation’ and ‘new’ derive from the same Latin verb Novare (‘to make new’).

6 The successful efforts made by the manufacturers of the DaVinci surgical robot to establish ‘substantial equivalence’ of their invention to existing technology is testament to this. See: [23].

7 For example, researchers in our own project involved in identifying the scope and frequency of SI would be aided by a definition; more widely, policy-makers devising mechanisms for external oversight of innovation also require such definitions; publishers, peer reviewers and grant awarding bodies may also find such definitions useful.

8 For a lengthier overview of major criticisms of classical theory see [41] pp 79–83.

9 This clearly speaks to Wittgenstein’s analogy of e.g. likening conceptual features to fibres in a thread: “we extend our concept … as in spinning a thread we twist fibre on fibre. And the strength of the thread does not reside in the fact that some one fibre runs through its whole length, but in the overlapping of many fibres” [80] §67.

10 For example, the ‘prototype’ paradigm relies on the notion of a conceptual core, but cannot adequately explain the core’s structure. The ‘exemplar’ paradigm cannot explain why some features of exemplars are taken to be representative of category properties while others are not. The ‘theory’ paradigm has difficulty explaining why one person’s theory of a concept should be comparable to another’s.

11 Within legal scholarship, Sinnott-Armstrong [74], from a similar standpoint, argues there are variety of types of definitions. Within his taxonomy his category of ‘precising definitions’ are definitions “that offer a precise use… linked to a practical purpose” (p 188). Rather than their accuracy, the success of such definitions, Sinnott-Armstrong maintains, is their utility to the particular purpose for which they are intended. We can thus see similarities with Sinnott-Armstrong’s work and our eliminativist position. Where we depart however, is that in our view that cleaving to the terms of a contested definition can cloud, rather than work for, the intended purpose.

12 The research protocol was registered with PROSPERO register of systematic reviews (ID CRD42017057492). The full protocol can be accessed at the following url: http://www.crd.york.ac.uk/PROSPERO/display_record.php?ID=CRD42017057492.

13 The full list of the sources is available from the corresponding author on request.

14 The claim from these authors utilises both wants (informed consent) and needs (no alternative), but prioritises the latter.

15 The possibility of a prudential benefit to society in allowing industry to make profits or surgical careers to flourish is a potential contradiction to this position. Our (anonymous) peer reviewer argues this seems a false dichotomy: surgical careers can flourish in the absence of large royalty and other payments from device manufacturers and most industries survive without the enormous profits generated in the biomedical field. (We are not committed to either position.)

16 The approach taken to geography will reflect whether innovation is subjectively or objectively defined, a question we broach below.

17 Identifying innovation as research may not produce clarity: Margo uses the term “informal” research [42], a term that apparently acknowledges the ambiguous status of both innovation and research.

18 This speaks to one of the conflicts of interest encountered by the subjective determination of SI favoured by the MSIIT.

19 Assuming, as this author does, that ‘experimental therapies’ equate to ‘innovation’.

20 They perhaps assume that innovation is done with appropriate arrangements and procedures in place.

21 For example, SI “differs from the standard technique” [30] p 951; “differs notably from the existing standard of care” [66]. Process differences that have been proposed to include patient time on anaesthesia [8]; different (additional) consent [70]; different technique, equipment, or patient group [30].

22 To a lesser extent the same could be true of the theme of “person” and “product”, given that intention plays a part in the analysis of both.

23 More general indications of risk and uncertainty, like ‘untested’ and ‘nonvalidated’ are also used to discuss SI, e.g. [7, 52].

24 This potentially collides definitions of innovation with definitions of research.

25 Me-too products are also found in medical innovation [51].

26 The necessity to reskill is flagged as a potential mark of SI suggesting that attention to the expertise of the surgeon distinguishes innovators from early adopters. See: [70].

27 Forssmann published the result of self-experiment in his first year after graduation. See: [60].

28 A subjective definition of ‘new to the individual’ is used by Everett Rogers in his theory of technological innovation [68].

29 We openly acknowledge here that our own normative concerns that give rise to this criticism are (1) there is insufficient monitoring and reporting of surgical practice, leading to potential for significant patient harm, and (2) overly restrictive governance has the potential to stifle beneficial developments in practice.

30 The figure is intended to be indicative, rather than exhaustive. Rather than summarise the implications of any or all of these claims, we refer the reader back to the full descriptions of the themes.

31 Indeed, an explicit aim of the IDEAL collaboration is to improve the reporting of SI, by facilitating a registry of operations that are first-in-human [46].

32 As entries are aggregated, convergence in reported criteria would potentially populate a fully fleshed description of SI that would inform multiple fields of inquiry.

33 The question could be raised about how our own definition will avoid these problems. This is a valid question (although not one that invalidates our critique). Briefly, our response is that not all concepts are as cluttered or contested as innovation, and thus there may be more common ground in a definition based on risk and sufficiency of knowledge. We plan to offer such arguments more thoroughly in a future worked explanation of our definition in a separate article.
